# Enhanced Cellular Immunity in Shrimp (*Litopenaeus vannamei*) after ‘Vaccination’

**DOI:** 10.1371/journal.pone.0020960

**Published:** 2011-06-16

**Authors:** Edward C. Pope, Adam Powell, Emily C. Roberts, Robin J. Shields, Robin Wardle, Andrew F. Rowley

**Affiliations:** 1 Centre for Sustainable Aquatic Research, Department of Biosciences, College of Science, Swansea University, Swansea, United Kingdom; 2 Intervet/Schering – Plough Animal Health (Aquaculture), Aquaculture Centre, Saffron Walden, United Kingdom; National Institute on Aging, United States of America

## Abstract

It has long been viewed that invertebrates rely exclusively upon a wide variety of innate mechanisms for protection from disease and parasite invasion and lack any specific acquired immune mechanisms comparable to those of vertebrates. Recent findings, however, suggest certain invertebrates may be able to mount some form of specific immunity, termed ‘specific immune priming’, although the mechanism of this is not fully understood (see Textbox S1). In our initial experiments, either formalin-inactivated *Vibrio harveyi* or sterile saline were injected into the main body cavity (haemocoel) of juvenile shrimp (*Litopenaeus vannamei*). Haemocytes (blood cells) from *V. harveyi*-injected shrimp were collected 7 days later and incubated with a 1∶1 mix of *V. harveyi* and an unrelated Gram positive bacterium, *Bacillus subtilis*. Haemocytes from ‘vaccinated’ shrimp showed elevated levels of phagocytosis of *V. harveyi*, but not *B. subtilis*, compared with those from saline-injected (non-immunised) animals. The increased phagocytic activity was characterised by a significant increase in the percentage of phagocytic cells. When shrimp were injected with *B. subtilis* rather than vibrio, there was no significant increase in the phagocytic activity of haemocytes from these animals in comparison to the non-immunised (saline injected) controls. Whole haemolymph (blood) from either ‘immunised’ or non-immunised’ shrimp was shown to display innate humoral antibacterial activity against *V. harveyi* that was absent against *B. subtilis*. However, there was no difference in the potency of antibacterial activity between *V. harveyi*-injected shrimp and control (saline injected) animals showing that ‘vaccination’ has no effect on this component of the shrimp's immune system. These results imply that the cellular immune system of shrimp, particularly phagocytosis, is capable of a degree of specificity and shows the phenomenon of ‘immune priming’ reported by other workers. However, in agreement with other studies, this phenomenon is not universal to all potential pathogens.

## Introduction

Invertebrates do not possess a specific, adaptive immune system based on the clonal expansion of activated lymphocytes, such as is found in vertebrates. In the absence of ‘true’ lymphocytes and functional antibody, they have been traditionally thought to rely instead entirely on innate (non-specific) immunity for internal defence against parasites and pathogens [1]. It has been argued that the cost limitation of a resource hungry, specific immune system may reduce other fitness related traits in invertebrates [2] and that the innate immune system is therefore the most ‘cost-effective’ method to defend these animals against disease. Recent reports, however, have suggested the existence of some form of immune memory in invertebrates that is referred to by some authors as ‘immune priming’ or ‘specific immune priming’ (e.g. [3], see Textbox S1). In certain cases, this enhanced resistance to infection appears to be passed from parents to progeny (so called ‘trans-generational immune priming’; [4]–[6]). It should be noted however, that some of these reports have attracted criticism (e.g. [7]) principally because they are largely based on phenomenological observations (e.g. increased resistance/survival following secondary challenge) and often lack mechanistic explanations for how the immune system accomplishes such heightened resistance to infection.

The studies of Watson et al. [8] have identified a potential mechanism for ‘specific immune priming’ in invertebrates. These workers found a homologue of the Down syndrome cell adhesion molecule (Dscam) in fruit flies (*Drosophila*) that is expressed in key phagocytic cell types in this insect's immune system. They also conclusively demonstrated that suppression of Dscam gene expression in flies by RNA interference, results in a marked reduction in the phagocytic activity of blood cells. Finally, they estimated that 18,000 isoforms of Dscam could be formed by sequence variation in the three immunoglobulin-like domains of this molecule and that these probably allowed Dscam to show differential binding to foreign agents in an analogous way to how vertebrate antibody binds different antigens. In a related invertebrate (the mosquito) further experimentation has revealed the production of different Dscam-like molecules with varying pathogen interaction specificities following challenge with different pathogens [9], again analogous to the clonal selection mechanism of vertebrate immunity. In turn, this modulated the phagocytic activities of the mosquito's haemocytes to enhance host survival following challenge. Such studies imply that following challenge with pathogens or parasites, variants of Dscam will be formed that enhance the clearance and destruction by phagocytic haemocytes of those invaders upon re-exposure. These Dscam homologues act as specific pattern recognition molecules that selectively bind microbes to the phagocytic blood cells. Hence Dscam is one of a number of candidates to explain the phenomenon of specific immune priming, at least in insects.

Little attention has been paid to the development of putative ‘vaccines’ aimed to boost growth and welfare in invertebrates because of their perceived lack of a specific immune system. Such approaches are, however, urgently needed for the aquaculture-based production of economically beneficial invertebrates such as shrimp and other decapods crustaceans. Over the last few years, global aquaculture has regularly produced over 2.4 million tonnes of cultured shrimp per annum, worth approximately $11 billion [10] and these values will probably increase with the projected rise in global population and associated increase in demand for protein [11]. Unfortunately, viral and bacterial disease pandemics, including vibriosis, have resulted in widespread shrimp mortality and significant economic loss over the past few decades [12].

The spread of disease may be prevented by controlling animal provenance, biosecurity and husbandry [12]. Nevertheless, there is a widespread and potentially damaging use of antibiotics and their residues can still enter the environment and human food chain [13]. More environmentally sustainable treatments, such as immune stimulants, probiotics and putative vaccines, could be an alternative method of promoting shrimp health without causing such problems. Therefore, there are continuing economic, ecological and public health incentives to explore the immune systems and immune modulators of these important crustaceans.

This study examines whether challenge with an inactivated bacterial shrimp pathogen, *Vibrio harveyi*, results in heightened immune reactivity in juvenile Pacific white shrimp (*Litopenaeus vannamei*). It also investigates the level of specificity of this response to ascertain if shrimp have any form of ‘specific’ or ‘selective’ immunity similar to that reported in insects [8], [9]

## Materials and Methods

### Animals

Pacific white shrimp, *Litopenaeus vannamei*, were raised from broodstock as described in detail elsewhere [14] and fed daily with a commercial diet (3% biomass d^−1^, Dragon Feeds Supreme™ shrimp diet). Rearing tanks were connected to a single, fully re-circulating system incorporating continuous mechanical and biological filtration, UV disinfection and temperature control of the system process water. Tank inlet water was kept at a temperature of 30°C and 30‰ salinity, and minimal, low concentrations of dissolved inorganic nitrogen were maintained.

### Experimental Design (see Figure 1)

**Figure 1:**
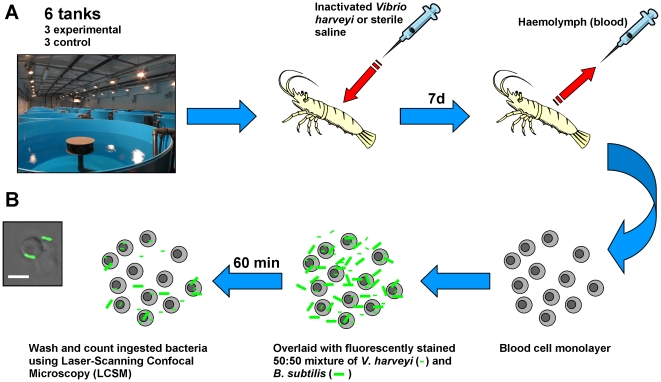
Experimental design for phagocytosis experiments. (A) Schematic diagram of experimental design. Juvenile shrimp (*L. vannamei*) were assigned randomly across 6×1500L tanks and injected with either formalin-inactivated *Vibrio harveyi* (a known shrimp pathogen; 10^8^ bacteria animal^−1^) or sterile 3% NaCl solution (12–14 animals tank^−1^, 3 tanks treatment^−1^). After 7 d, monolayers of shrimp haemocytes were overlaid with a mixture of fluorescently-labelled formalin-inactivated *V. harveyi* and an unrelated bacterium (*Bacillus subtilis*; 50∶50 mixture). The ability of the haemocytes to phagocytose the different bacteria was then investigated using LSCM. (B) High-power LSCM projection of 15 optical sections through a shrimp haemocyte (total *z*-axis = 10.4 µm) and four FITC-labelled *Bacillus subtilis* bacteria. Whether an individual bacteria is internalised or not can be determined through sequential viewing of each optical section. Scale bar = 5 µm.

In Experiment 1, juvenile *L. vannamei* weighing 17.4±4.1 g (mean ±1 S.D., *N* = 80) were injected with either 100 µl of formalin-inactivated *Vibrio harveyi* (VIB 645 CZV-STL-1; 10^8^ bacteria animal^−1^) or 100 µl of filter sterilised (0.22 µm) 3% NaCl solution. This strain of *V. harveyi* was supplied by Intervet/Schering – Plough Animal Health and is known to be pathogenic to shrimp. Experimental groups were assigned randomly across 6×1500L tanks (12–14 animals tank^−1^, 3 tanks treatment^−1^) and monitored for moulting and mortality twice daily, with all moults and dead animals removed. Animals were sacrificed for the phagocytosis assay and antibacterial assay 7 and 8 d post-injection (post-vaccination) respectively. The sterility of the formalin-inactivated *V. harveyi* and saline was verified by plating on tryptic soy agar (+2% NaCl) and incubating at 25°C for 48 h. Whole haemolymph (plasma and blood cells) for the antibacterial assay was obtained by bleeding from the ventral blood vessel of anaesthetised (30 s on ice) animals using a sterile 21G needle and immediately stored at −80°C. Previous work in our laboratory has shown that freezing does not impair antibacterial potency when compared to preliminary experiments using fresh tissue (data not shown).

Experiment 2 used the same methodology as the first trial except juvenile shrimp (11.5±1.4 g, mean ±1 S.D., *N* = 41) were injected with formalin-inactivated *B. subtilis* (10^8^ bacteria animal^−1^) or the same volume of sterile 3% NaCl solution, with a second injection of the same treatment 7 d later. Animals were sacrificed for the phagocytosis assay 7 d after the second injection (i,e, Day 14).

### Phagocytosis Studies

#### Preparation of target bacteria

*Bacillus subtilis* (NCIMB 1048; originally isolated from the marine environment) and *V. harveyi* (VIB 645 CZV-STL-1) were each grown overnight in TSB (+2% NaCl) with constant agitation, centrifuged (4500 **g**, 4°C, 10 min) and washed three times in filter sterilised (0.22 µm) marine saline (0.5 M NaCl, 12 mM CaCl_2_·2H_2_O, 11 mM KCl, 26 mM MgCl_2_·6H_2_O, 50 mM Tris; pH 7.4). The resulting bacterial suspensions were taken through 21, 26 and 27G needles to disrupt any chains and formalin-inactivated (2% formaldehyde, overnight, 4°C). The suspensions were then washed extensively in sterile marine saline and counted. The sterility of the formalin-inactivated bacterial suspensions was verified by plating on tryptic soy agar (+2% NaCl).

Formalin-inactivated *V. harveyi* or *B. subtilis* suspensions, both containing 2.5×10^9^ bacteria, were washed three times with sterile marine saline (4500 **g**, 4°C, 10 min). The two suspensions were combined in 1 ml 0.2 M carbonate-bicarbonate buffer (pH 9.4) at a 1∶1 ratio before 0.1 mg (0.1 mg ml^−1^) fluorescein isothiocyanate (FITC) was added and the suspension was incubated in the dark on a rotator (30 min, RT). The bacteria were washed three times in carbonate-bicarbonate buffer and re-suspended in 20 ml sterile marine saline (100 µl contained 2.5×10^7^ of each bacteria). The suspension of labelled bacteria was then divided into 1 ml aliquots and stored at −20°C for later use.

For Experiment 2, the target bacteria were prepared as described previously but a mixture of formalin-inactivated *Vibrio* spp. (*V. anguillarum* biotype I and II, *V. harveyi*, *V. parahaemolyticus* and *V. vulnificus*) was used instead of just *V. harveyi* (ratio *B. subtilis*: *Vibrio* spp. = 1∶1)

#### Phagocytosis assays

Shrimp were anaesthetised by placing on ice for *ca.* 30 s. The ventral sinus was pierced using a wide-bore (19G) needle and the haemolymph collected in a Petri-dish containing 5 ml ice-cold marine anticoagulant (MAC; 0.45 M NaCl, 0.1 M glucose, 30 mM trisodium citrate, 26 mM citric acid, 10 mM EDTA; pH 4.6; modified from [15], [16]) over ice. The contents of the dish were transferred to 20 ml ice-cold MAC and centrifuged (2000 **g**, 10 min, 4°C). The haemocytes were re-suspended in 5 ml ice-cold MAC and washed, counted and re-suspended in a volume of sterile marine saline so that 100 µl contained 5×10^5^ cells. A Teflon hydrophobic pen (Sigma-Aldrich) was used to mark rectangular areas *ca.* 10×5 mm on slides that had been washed with distilled water, ethanol and distilled water again. Cell suspension (100 µl) was then dispensed into each marked area and the slides were incubated to allow the haemocytes to adhere (20 min, 25°C). The saline was then drained and replaced with 100 µl of the labelled bacteria solution (final cell∶bacteria ratio = 1∶50) and incubated in the dark (1 hr, 25°C). The slides were rinsed in marine saline and fixed in 5% Baker's fixative (2 hr; 5% formalin, 1% calcium acetate in marine saline, by volume) before being air-dried overnight and stored dry in the dark. The slides were mounted using Permafix HRF (Citifluor Ltd., Leicester, UK) and stored at 4°C for examination using a Zeiss LSM 510 META laser scanning confocal microscope. The number of phagocytosed bacteria of each type was recorded per cell. Counts were converted to the number of bacteria phagocytosed per 100 haemocytes. The percentage of cells that were phagocytic was also calculated and a phagocytic index, which determined the average number of bacteria per phagocyte, was calculated according to the formula:
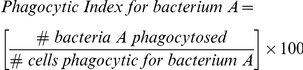



Discrimination between intra- and extra-cellular bacteria was achieved using a series of optical sections along the z axis. By viewing these sections in sequence, it was possible to determine if bacteria were internalised or simply attached to the cell surface. Similarly, the Gram positive *B. subtilis* could clearly be differentiated from the Gram negative *Vibrio* spp. because of their larger size.

### Antibacterial assay

The assay used to investigate levels of humoral antibacterial activity of whole haemolymph was based on that described previously [17], [18]. Whole haemolymph samples were thawed over ice, taken through sterile 19G needles to disrupt cellular material and centrifuged (500 **g**, 10 min, 4°C) to produce a supernatant for immediate use in the antibacterial assay. The sterility of this supernatant was verified by plating 100 µl on tryptic soy agar and incubating at 25°C for 48 hr in triplicate.

*V. harveyi* (VIB 645 CZV-STL-1) and *B. subtilis* (NCIMB 1048) were grown overnight (12 hr, 25°C) in tryptic soy broth (TSB) supplemented with 2% NaCl. After washing twice in sterile 3% NaCl solution (4500 **g**, 10 min, 4°C), 1 ml was taken, passed through a 27G needle to disrupt any chains and counted. The bacteria were then re-suspended in a volume of sterile 3% NaCl solution calculated to give 10^8^ bacteria ml^−1^ and stored on ice.

For the antibacterial assay, 50 µl of bacteria (0.5×10^7^ bacteria) and 50 µl haemolymph supernatant, or an equal volume of sterile 3% NaCl solution, were pipetted into each well of a sterile, flat-bottom 96 well plate and incubated at 20°C for 30 min with shaking. Subsequently, 50 µl of this suspension was added to a further sterile 96 well plate, 200 µl of TSB containing 2% NaCl (final concentration) added and the plate incubated at 25°C for 24–48 h on a plate reader with the absorbance at 550 nm measured each hour. Bacteria incubated with sterile 3% NaCl solution were used as a bacteria-only control'. The values at T = 0 were subtracted for each well before nonlinear regression was used to calculate a T_50_ value for each treatment as follows:
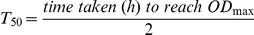
Where OD_max_ = maximum OD_550_ absorbance obtained with the bacteria-only control. To compare each treatment, data for the time point closest to the T_50_ value for each strain's bacteria-only control were analysed statistically.

### Statistical Analysis

All data were analysed using GraphPad Prism v. 5 for Windows (GraphPad Software, San Diego, USA) and SPSS v.13.0. Survival of juvenile *L. vannamei* post-injection was compared using Kaplan-Meier survival analysis with pair-wise comparisons. Results from the phagocytosis experiments were compared using 2-way ANOVA with proportional (% phagocytosis) data arcsin (sqrt) transformed prior to analysis. For the antibacterial assay experiments, the nonlinear regression function of GraphPad Prism was used calculate T_50_ values and data for individual time points were analysed using 1-way ANOVA.

## Results

### Survival of shrimp post-‘vaccination’

In Experiment 1, the shrimp in one tank showed significantly reduced survival after injection with formalin-inactivated *V. harveyi* compared with all other thanks (41.7% surviving after 7 d, p<0.05, Kaplan-Meier survival analysis with pair-wise comparisons, see Table S1) and so animals from this tank were not used in any studies. The remaining 5 tanks (3 saline-injected and 2 *V. harveyi* injected) showed high survival (92.3–100% survival after 7 d) with no significant differences between tanks or treatments (Kaplan-Meier survival analysis).

### Changes in phagocytic activity of haemocytes in juvenile shrimp following injection of inactivated bacteria

**Figure 2:**
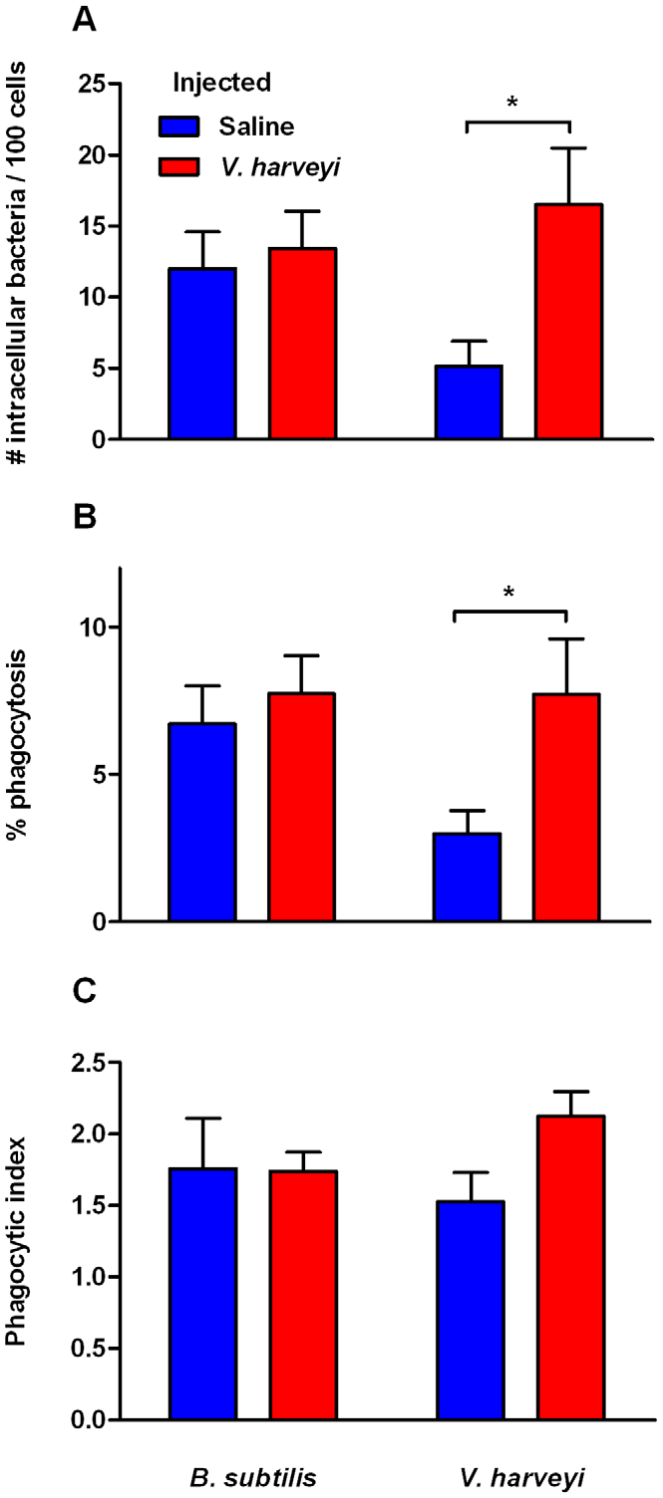
Elevation of phagocytic activity in juvenile shrimp blood cells following injection of inactivated bacteria. Juvenile *Litopenaeus vannamei* were injected with 100 µl of either formalin-killed *Vibrio harveyi* or sterile saline. After 7 d, monolayers of shrimp haemocytes were overlaid with a 50∶50 mix of formalin-inactivated *V. harveyi* or an unrelated bacterium (*Bacillus subtilis*) and the number of phagocytosed bacteria of each type was recorded per cell. (A) Number of intracellular bacteria per 100 haemocytes, (B) percentage of haemocytes that showed phagocytic activity and (C) phagocytic indices (a measure of the number of bacteria internalised per phagocytic cell). * p<0.05, 2-way ANOVA with Bonferroni post-test. Mean ±1 S.E., *N* = 8–9.

In Experiment 1 shrimp previously injected with formalin-inactivated *V. harveyi* showed significantly more fluorescently-labelled *V. harveyi* per 100 haemocytes (blood cells) than those injected with sterile saline (*p*<0.05, 16.50±3.98 *vs.* 5.18±1.75 respectively, mean values ±1 S.E.M.; Figure 2A). Similarly, the percentage of haemocytes phagocytosing *V. harveyi* was significantly higher in animals previously injected with *V. harveyi* than those injected with sterile saline (p<0.05, 7.72±1.88 *vs.* 3.00±0.77, mean ±1 S.E.M.; Figure 2B). There were no significant differences between treatments for the number of bacteria per 100 cells or percentage phagocytosis of *B. subtilis*. Finally, there were no significant differences in the phagocytic indices (a measure of the number of bacteria internalised per phagocytic cell) between treatments for either bacterium (Figure 2C).

In Experiment 2, shrimp were ‘vaccinated’ with *B. subtilis* rather than *V. harveyi*, with a second injection after 7 d. Changes in phagocytic activity of haemocytes were recorded 7 d after the second vaccination (Day 14). Unlike the case with *V. harveyi*, as seen in Experiment 1, previous exposure to *B. subtilis* failed to cause any elevation in the number of bacteria per 100 cells, the percentage of phagocytic cells or the phagocytic indices for either *B. subtilis* or the mix of *Vibrio* spp. Indeed, there was a significant reduction in the number of phagocytosed *B. subtilis* per 100 cells (p<0.01, 13.89±1.72 *vs.* 26.78±2.98, mean ±1 S.E., *N = 5–8*) and percentage of phagocytic cells (p<0.01, 9.76±1.08 *vs.* 17.28±1.62, mean ±1 S.E., *N* = 5–8). Previous injection with *B.* subtilis had no effect on the phagocytic index for *B. subtilis* or the number of bacteria per 100 cells, percentage phagocytic cells or phagocytic index for the mix of vibrios (data not shown).

### Changes in antibacterial activity of shrimp blood following *V. harveyi* exposure

**Figure 3:**
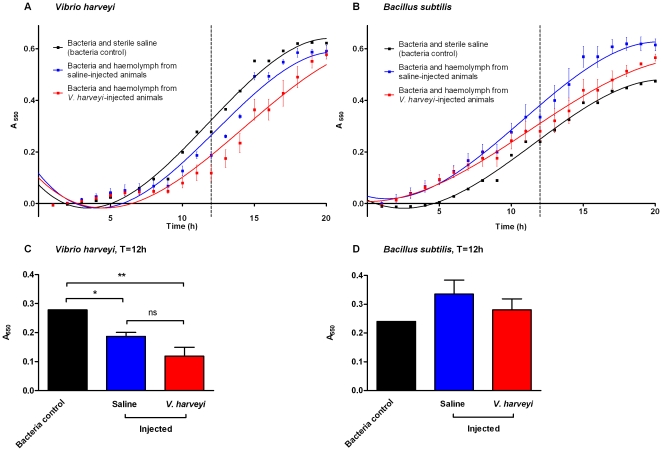
Antibacterial activity of shrimp blood following *V. harveyi* exposure remains unchanged. Juvenile shrimp (*Litopenaeus vannamei*) were injected with 100 µl of either formalin-inactivated *Vibrio harveyi* or sterile saline. After 8 d, shrimp haemolymph was added to either *V. harveyi* or an unrelated, Gram-positive bacterium, *Bacillus subtilis*. (A) Growth curves for *V. harveyi* and (B) *B. subtilis* exposed to shrimp haemolymph from animals injected with either sterile saline or inactivated *V. harveyi*, or sterile NaCl solution (‘bacteria-only control’ i.e. no haemolymph). Bacterial growth was recorded as absorbance at 550 nm after T = 0 had been subtracted. (C) and (D) experimental data from the time (h) closest to the calculated T_50_ for the bacteria-only control (dotted lines in figs. 3a & B) of each bacterial species (see Table S2). ns = no significant difference, * p<0.05, ** p<0.01 compared with bacteria control (1-way ANOVA with Bonferroni post-test). Mean ±1 S.E., *N* = 3.

The growth of *V. harveyi* exposed to shrimp haemolymph was impeded compared to the bacteria-only control (i.e. bacteria and sterile saline; see figure 3A) and the T_50_ values (see Table S2) were considerably higher, suggesting antibacterial activity. Data for the time-point closest to the calculated bacterial-only control T_50_ value (see Table S2) were further analysed (Figure 3C) and shrimp blood was found to show significant natural antibacterial activity against *V. harveyi* (p<0.01) that did not differ between *V. harveyi*-injected and saline-injected animals. No antibacterial activity was observed in the haemolymph of shrimp against *B. subtilis* regardless of their vaccination status (Figures 3B and D).

## Discussion

In this study we have demonstrated that previous exposure of juvenile shrimp to a formalin-inactivated pathogenic vibrio results in the increased phagocytic uptake of this bacterium by the haemocytes. The phagocytosis of an unrelated Gram positive bacterium, *Bacillus subtilis*, by the same haemocytes was unchanged compared to cells taken from shrimp injected with saline. This suggests the stimulation of phagocytic activity observed towards *V. harveyi* is selective compared with *B. subtilis*. Furthermore, our results suggest that increased phagocytosis largely results from an increase in the percentage of cells that are phagocytic, rather than an increase in the activity of resident phagocytes.

Our results agree with those of another study using the American lobster (*Homarus americanus*) that observed enhanced phagocytosis of the bacterium, *Aerococcus viridans* var *homari* (the causative agent of the lobster disease gaffkaemia) after challenge with the same species [19]. In addition, these workers also observed a degree of specificity as there was no increase in phagocytosis of an unrelated bacterium that was not in the ‘vaccine’. The results of the current study also concur with those of a recent study where shrimp (*L. vannamei*) were immunised with a commercial vaccine (Vibromax™; a mixture of formalin-killed vibrios) and the phagocytic activity of haemocytes taken from immunised and non-immunised animals compared [18].

Fitzgerald and Ratcliffe [20] were the first workers to report that invertebrate phagocytes recognise Gram positive and Gram negative bacteria by separate mechanisms, presumably because of the distinct cell wall structures of these microbes. This specificity may arise from pattern recognition proteins (PRPs) either in the haemolymph or directly associated with the phagocytic haemocytes that bind pathogen-associated molecular patterns on different microbial surfaces (PAMPs; [1]) but our knowledge of these PRPs in invertebrates is still rudimentary. There may be distinct PRPs in invertebrates for PAMPS found in Gram positive and Gram negative bacteria. It is possible that the cellular response following exposure of the shrimp immune system to formalin-inactivated *V. harveyi* forms PRPs that are specific or selective for Gram negative bacteria only. These subsequently bind to *V. harveyi* in the phagocytosis assay resulting in their heightened internalisation and perhaps intracellular killing. Alternatively, the shrimp immune system may be able to synthesise PRPs specific to a particular bacterium. Either hypothesis would enable these animals to deal more vigorously with later chance exposure to potentially pathogenic bacteria, with the hallmarks of apparent memory and specificity that characterises the vertebrate immune system.

There are several candidates for PRPs in *L. vannamei* including Dscam and lectins. The role of Dscam in insect immunity is increasingly clear where it appears to form a large number of variants each with their own differential ability to bind to various parasites and pathogens. However, whilst homologues of Dscam have been identified in a number of crustaceans including the water flea, *Daphnia* [21], *L. vannamei* [22] and another shrimp, *Penaeus monodon* [23], functional studies equivalent to those in insects [e.g. 8], [9] are lacking. A Dscam-like protein (LVDscam) has recently been isolated and characterised from *L. vannamei* [22]. This form is dissimilar to that of insects studied to date in that it lacks a characteristic cytoplasmic tail and transmembrane domain that are involved in membrane binding. However, in another closely related shrimp, *P. monodon*, one of the Dscam homologues identified has a cytoplasmic tail which the authors suggest may allow it to bind to the membranes of haemocytes and hence act as a putative antigen receptor [23]. The Dscam gene identified from *Daphnia* spp. has been shown to have the potential to form over 13,000 different protein isomorphs [21] which may possess differential ability to bind PAMPs and hence act as a variable recognition molecule. A second candidate for PRP in shrimp are lectins which are widely distributed in invertebrates and have been shown to act as recognition molecules [24], [25]. Recent studies have shown that one lectin, the fibrinogen-related protein found in the snail, *Biomphalaria glabrata* exhibits structural diversity in response to parasites [26], [27]. Lectins are also found in shrimp haemolymph [28] but whether they have the same potential for diversity of PAMP binding as seen in the snail is unknown..

An interesting observation of the current study is that while ‘vaccination’ of shrimp with *V. harveyi* apparently yields greater levels of haemocyte mediated phagocytic activity, the same experiment repeated with *B. subtilis* failed to show the same stimulation. This questions the universality of specific ‘immune priming’ as a phenomenon in shrimp and perhaps invertebrates in general. This has also been found to be the case in other studies of immune priming in invertebrates. For instance, Roth et al. [3] in their studies on changes in the resistance of red flour beetles (*Tribolium castaneum*) following vaccination with *Bacillus thuringiensis*, *B. subtilis* or *Escherichia coli*, found that while enhanced resistance emerged as a result of vaccination with *B. thuringiensis*, in the case of *E. coli* such a phenomenon could not be achieved. Their suggestion for the difference in response was that *B. thuringiensis* is a natural pathogen of insects and hence such animals would commonly encounter this organism and require defences designed to provide protection. In our current study, *V. harveyi* is known to be a pathogen of shrimp and vibrios are widely found in the aquatic environment [29] while *B. subtilis*, a member of the Bacillaceae, is not normally considered to inhabit this environment. Overall, these reports suggest that the phenomenon of specific ‘immune priming’ may not be universal for all pathogens and parasites. Indeed, it is possible that the observation of immune priming in our study is unique to *V. harveyi*, and not a general property of shrimp immunity.

This study also demonstrated that shrimp haemolymph shows natural antibacterial activity against *Vibrio harveyi* but that this activity is unaffected by prior exposure (i.e. ‘vaccination’). This implies that ‘vaccination’ of shrimp with *V. harveyi* fails to induce any demonstrable changes in levels of antibacterial factors at least with the doses injected and timescale examined. Previous studies have found a range of constitutively expressed antimicrobial peptides/proteins in shrimp including crustins and penaeidins [30]–[32]. Whilst some workers have demonstrated elevated levels of humoral antibacterial activity in invertebrates that manifest the phenomenon of ‘immune priming’ (e.g. [6]), our results support the view that it is doubtful whether the specificity of action of immune peptides, such as the defensin family, that act as broad spectrum antimicrobial agents, could be responsible for such observations (see [1] for a detailed discussion).

### Concluding Remarks

There has been much debate in the last few years about the possibility that invertebrates are able to mount an immune response with some of the hallmarks of the vertebrate immune system (i.e. memory and specificity). Two views exist, those who fail to be convinced that the phenomenological studies prove the existence of any form of immunological memory such as ‘specific immune priming’ (e.g. [7]) and others (e.g. [8], [9], [18], [33], [34]) who have found potential mechanistic explanations that could account for some initial purely phenomenological observations that claimed the existence of ‘specific immune priming’. The current study has demonstrated heightened phagocytic activity after bacterial challenge that is not present with an unrelated bacterium. Whether vibrio-primed shrimp are more able to clear and eliminate such bacteria than naïve animals needs to be ascertained, as does the temporal nature of this stimulation. The universality of specific immune priming is questioned by our observations that immunisation with another bacterial species (*B. subtilis*) does not result in heightened cellular (i.e. phagocytosis) immunity.

## Supporting Information

Table S1 **Survival of experimental animals.** Percentage survival of juvenile *L. vannamei* 7 d after injection with formalin-inactivated *V. harveyi* or sterile saline. Kaplan-Meier survival analysis with pair-wise comparisons against all other tanks.
(DOCX)

Table S2 **LT_50_ values for bacteria exposed to haemolymph from ‘vaccinated’ shrimp.** T_50_ values (h) for *V. harveyi* and *B. subtilis* exposed to haemolymph from *L. vannamei* previously injected with formalin-inactivated *V. harveyi* or sterile saline. A T_50_ value less than that calculated for the bacteria grown with sterile NaCl solution (bacterial control, i.e. no haemolymph) indicates more rapid growth whilst a greater value suggests impeded growth (possible antibacterial activity). 95% Confidence Intervals in parentheses.
(DOCX)

Textbox S1 **Defining adaptive immunity in invertebrates.**
(DOCX)

## References

[1] Rowley AF, Powell A (2007). Invertebrate immune systems–specific, quasi-specific, or nonspecific?. J Immunol.

[2] Schmid-Hempel P (2005). Evolutionary ecology of insect immune defences.. Annu Rev Entomol.

[3] Roth O, Sadd BM, Schmid-Hempel P, Kurtz J (2009). Strain-specific priming of resistance in the red flour beetle, *Tribolium castaneum*.. Proc R Soc B.

[4] Little TJ, O'Connor B, Colegrave N, Watt K, Read AF (2003). Maternal transfer of strain-specific immunity in an invertebrate.. Current Biol.

[5] Sadd BM, Heimpel Y, Schmid-Hempel R, Schmid-Hempel P (2005). Trans-generational immune priming in a social insect.. Biol Lett.

[6] Moret Y (2006). ‘Trans-generational immune priming’: specific enhancement of the antimicrobial immune response in the mealworm beetle, *Tenebrio molitor*.. Proc R Soc B.

[7] Hauton C, Smith VJ (2007). Adaptive immunity in invertebrates: a straw house without a mechanistic foundation.. Bio Essays.

[8] Watson FL, Püttmann-Holgado R, Thomas F, Lamar DL, Hughes M (2005). Extensive diversity of Ig-superfamily proteins in the immune system of insects.. Science.

[9] Dong Y, Taylor HE, Dimopoulos G (2006). AgDscam, a hypervariable immunoglobulin domain-containing receptor of the *Anopheles gambiae* innate immune system.. PLoS Biol.

[10] FAO (2006). ftp://ftp.fao.org/docrep/fao/009/a0874e/a0874e00.pdf.

[11] Garcia SM, Grainger RJR (2005). Gloom and doom? The future of marine capture fisheries.. Phil Trans R Soc Lond B.

[12] Lightner DV (2005). Biosecurity in shrimp farming: pathogen exclusion through use of SPF stock and routine surveillance.. J World Aquac Soc.

[13] Cabello FC (2006). Heavy use of prophylactic antibiotics in aquaculture: a growing problem for human and animal health and for the environment.. Environ Microbiol.

[14] Diamond S, Powell A, Shields RJ, Rowley AF (2008). Is spermatophore melanisation in captive shrimp (*Litopenaeus vannamei*) a result of an auto-immune response?. Aquaculture.

[15] Söderhäll K, Smith V (1983). Separation of the haemocyte populations of *Carcinus maenas* and other marine decapods, and prophenoloxidase distribution.. Dev Comp Immunol.

[16] Vargas-Albores F, Guzmán M-A, Ochoa J-L (1993). An anticoagulant solution for haemolymph collection and prophenoloxidase studies of penaeid shrimp (*Penaeus californiensis*).. Comp Biochem Physiol A.

[17] Bexfield A, Nigam Y, Thomas S, Ratcliffe NA (2004). Detection and partial characterisation of two antibacterial factors from the excretions/secretions of the medicinal maggot *Lucilia sericata* and their activity against methicillin-resistant *Staphylococcus aureus* (MRSA).. Microb Infect.

[18] Powell A, Pope EC, Eddy FE, Roberts EC, Shields RJ (2011). Enhanced immune defences in Pacific white shrimp (*Litopenaeus vannamei*) post-exposure to a vibrio vaccine.. J Invertebr Pathol.

[19] Mori K, Stewart JE (2006). Immunogen-dependent quantitative and qualitative differences in phagocytic responses of the circulating hemocytes of the lobster *Homarus americanus*.. Dis Aquat Org.

[20] Fitzgerald SW, Ratcliffe NA (1982). Evidence for the presence of subpopulations of *Arenicola marina* coelomocytes identified by their selective response towards Gram+ve and Gram-ve bacteria.. Dev Comp Immunol.

[21] Brites D, McTaggart S, Morris K, Anderson J, Thomas K (2008). The Dscam homologue of the crustacean *Daphnia* is diversified by alternative splicing like in insects.. Mol Biol Evol.

[22] Chou P-H, Chang H-S, Chen IT, Lin H-Y, Chen Y-M (2009). The putative invertebrate adaptive immune protein *Litopenaeus vannamei* Dscam (LvDscam) is the first reported Dscam to lack a transmembrane domain and cytoplasmic tail.. Dev Comp Immunol.

[23] Chou P-H, Chang H-S, Chen I-T, Lee C-W, Hung H-Y (2011). *Penaeus monodon* Dscam (PmDscam) has a highly diverse cytoplasmic tail and is the first membrane-bound shrimp Dscam to be reported.. Fish Shellfish Immunol.

[24] Loker ES, Adema CM, Zhang SM, Kepler TB (2004). Invertebrate immune systems – not homogeneous, not simple, not well understood.. Immunol Rev.

[25] Ghosh J, Lun CM, Majeske AJ, Sacchi S, Schrankel CS (2011). Invertebrate immune diversity.. Dev Comp Immunol.

[26] Zhang SM, Adema CM, Kepler TB, Loker ES (2004). Diversification of Ig superfamily genes in an invertebrate.. Science.

[27] Moné Y, Gorbal B, Duval D, Du Pasquier L, Kieffer-Jaquinod S (2010). A large repertoire of parasite epitopes matched by a large repertoire of host immune receptors in an invertebrate host/parasite model.. PLoS Negl Trop Dis.

[28] Zhang X-W, Xu W-T, Wang X-W, Mu Y, Zhao X-F (2009). A novel C-type lectin with two CRD domains from Chinese shrimp *Fenneropenaeus chinensis* functions as a pattern recognition protein.. Mol Immunol.

[29] Manilal A, Sujith S, Selvin J, Shakir C, Gandhimathi G (2010). Virulence of vibrios isolated from diseased black tiger shrimp, *Penaeus monodon*, Fabricius.. J World Aquaculture Soc.

[30] Muňoz M, Vandenbulke F, Gueguen Y, Bachère E (2003). Expression of penaeidin antimicrobial peptides in early larval stages of the shrimp *Penaeus vannamei*.. Dev Comp Immunol.

[31] Jiravanichpaisal P, Puanglarp N, Petkon S, Donnuea S, Söderhäll I (2007). Expression of immune-related genes in larval stages of the giant tiger shrimp, *Penaeus monodon*.. Fish Shellfish Immunol.

[32] Muňoz M, Vandenbulcke F, Saulnier D, Bachère E (2002). Expression and distribution of penaeidin antimicrobial peptides are regulated by haemocyte reactions in microbial challenged shrimp.. Eur J Biochem.

[33] Pham LN, Dionee MS, Shirasu-Hiza M, Schneider DS (2007). A specific primed immune response in *Drosophila* is dependent on phagocytes.. PLoS Pathogens.

[34] Roth O, Kurtz J (2010). Phagocytosis mediates specificity in the immune defence of an invertebrate, the woodlouse *Porcellio scaber* (Crustacea: Isopoda).. Dev Comp Immunol.

