# Supplementary material for: Enhanced Cellular Immunity in Shrimp (Litopenaeus vannamei) after ‘Vaccination’
Source: PLoS One. 2011 Jun 16;6(6):e20960. doi: 10.1371/journal.pone.0020960 (PMC3116845; doi:10.1371/journal.pone.0020960)
Supplement: Table S1 — Survival of experimental animals. Percentage survival of juvenile L. vannamei 7 d after injection with formalin-inactivated V. harveyi or sterile saline. Kaplan-Meier survival analysis with pair-wise comparisons against all other tanks. (DOCX) [file pone.0020960.s001.docx]

| **Tank** | **Treatment** | **N** | **Deaths** | **% Survival** | **P** |
| --- | --- | --- | --- | --- | --- |
| 1 | *V. harveyi* | 12 | 7 | 41.67 | <0.05 |
| 2 | Saline | 13 | 1 | 92.31 | ns |
| 3 | Saline | 14 | 0 | 100.00 | ns |
| 4 | *V. harveyi* | 13 | 0 | 100.00 | ns |
| 5 | *V.harveyi* | 14 | 1 | 92.76 | ns |
| 6 | Saline | 14 | 0 | 100.00 | ns |
| **Total** |  | **80** | **9** | **87.81** |  |
