# Supplementary material for: Enhanced Cellular Immunity in Shrimp (Litopenaeus vannamei) after ‘Vaccination’
Source: PLoS One. 2011 Jun 16;6(6):e20960. doi: 10.1371/journal.pone.0020960 (PMC3116845; doi:10.1371/journal.pone.0020960)
Supplement: Table S2 — LT50 values for bacteria exposed to haemolymph from ‘vaccinated’ shrimp. T50 values (h) for V. harveyi and B. subtilis exposed to haemolymph from L. vannamei previously injected with formalin-inactivated V. harveyi or sterile saline. A T50 value less than that calculated for the bacteria grown with sterile NaCl solution (bacterial control, i.e. no haemolymph) indicates more rapid growth whilst a greater value suggests impeded growth (possible antibacterial activity). 95% Confidence Intervals in parentheses. (DOCX) [file pone.0020960.s002.docx]

|  | ***V. harveyi*** | ***B. subtilis*** |
| --- | --- | --- |
| Sterile saline (bacteria-only control) | 12.07 (11.67-12.45) | 11.94 (11.56-12.30) |
| Haemolymph from *L. vannamei* injected with sterile saline | 13.33 (13.03-13.62) | 9.03 (8.56-9.48) |
| Haemolymph from *L. vannamei* injected with inactivated *V. harveyi* | 15.08 (14.77-15.37) | 10.04 (9.55-10.50) |
