# Supplementary material for: Enhanced Cellular Immunity in Shrimp (Litopenaeus vannamei) after ‘Vaccination’
Source: PLoS One. 2011 Jun 16;6(6):e20960. doi: 10.1371/journal.pone.0020960 (PMC3116845; doi:10.1371/journal.pone.0020960)
Supplement: Textbox S1 — Defining adaptive immunity in invertebrates. (DOCX) [file pone.0020960.s003.docx]

It is well known that invertebrates lack the cellular and molecular machinery that enables them to respond to infection by the formation of specific antibodies. In the last decade a number of publications have shown that invertebrates have a form of adaptive immunity that is referred to as ‘immune priming’ [4-6] or ‘alternative adaptive immunity’ [23]. Roth and co-workers [3] have defined immune priming as “*lasting immunity following an initial exposure that proves protection on a secondary exposure*”. In those cases where the immunity shows antigen specificity in terms of elevation in protection, this event has been defined as ‘specific immune priming’. For example, if an organism is exposed to pathogen ‘A’ and later exposed to the same pathogen (A) and an unrelated form (pathogen ‘B’) only heightened immunity or protection will be shown against ‘A’ and not ‘B’. This level of specificity may be highly specific such that different strains of the same microbial species may be differentiated [34] implying a high level of discriminatory ability by the immune systems of some invertebrates.
